# Mental Health Mobile Apps for Preadolescents and Adolescents: A Systematic Review

**DOI:** 10.2196/jmir.7332

**Published:** 2017-05-25

**Authors:** Rebecca Grist, Joanna Porter, Paul Stallard

**Affiliations:** ^1^ Child and Adolescent Mental Health Group Department for Health University of Bath Bath United Kingdom; ^2^ Child and Family Mental Health Temple House Oxford Health National Health Service Foundation Trust Keynsham United Kingdom

**Keywords:** mobile apps, smartphone apps, mHealth, mental health, self-help, child, adolescent, preadolescent, review

## Abstract

**Background:**

There are an increasing number of mobile apps available for adolescents with mental health problems and an increasing interest in assimilating mobile health (mHealth) into mental health services. Despite the growing number of apps available, the evidence base for their efficacy is unclear.

**Objective:**

This review aimed to systematically appraise the available research evidence on the efficacy and acceptability of mobile apps for mental health in children and adolescents younger than 18 years.

**Methods:**

The following were systematically searched for relevant publications between January 2008 and July 2016: APA PsychNet, ACM Digital Library, Cochrane Library, Community Care Inform-Children, EMBASE, Google Scholar, PubMed, Scopus, Social Policy and Practice, Web of Science, Journal of Medical Internet Research, Cyberpsychology, Behavior and Social Networking, and OpenGrey. Abstracts were included if they described mental health apps (targeting depression, bipolar disorder, anxiety disorders, self-harm, suicide prevention, conduct disorder, eating disorders and body image issues, schizophrenia, psychosis, and insomnia) for mobile devices and for use by adolescents younger than 18 years.

**Results:**

A total of 24 publications met the inclusion criteria. These described 15 apps, two of which were available to download. Two small randomized trials and one case study failed to demonstrate a significant effect of three apps on intended mental health outcomes. Articles that analyzed the content of six apps for children and adolescents that were available to download established that none had undergone any research evaluation. Feasibility outcomes suggest acceptability of apps was good and app usage was moderate.

**Conclusions:**

Overall, there is currently insufficient research evidence to support the effectiveness of apps for children, preadolescents, and adolescents with mental health problems. Given the number and pace at which mHealth apps are being released on app stores, methodologically robust research studies evaluating their safety, efficacy, and effectiveness is promptly needed.

## Introduction

Mental health problems are common in children and young people. Prevalence data suggests that up to 20% of children and young people up to 18 years of age have a diagnosable mental health problem [1,2]. Mental health problems in children persist with 50% of mental illness in adulthood beginning before the age 15 years and 75% before the age of 18 years [3]. Mental health problems cause significant distress and negatively impact on social relationships, school and occupational attainment, and physical health [4]. They also increase the risk of developing other mental health problems in adulthood [5]. Although evidence-based interventions are available for many child mental health problems, treatment services are limited and many children and adolescents either cannot or do not access appropriate help [2,4].

Digital technology provides a way of increasing access to evidence-based interventions [6]. Computerized cognitive behavioral therapy (CBT), for example, offers a promising and acceptable way of delivering interventions for anxiety and depression for children and young people [6,7]. However, technology is constantly evolving and mobile technologies in particular are being adopted at an increasing rate; by 2020, it is estimated there will be 6.1 billion mobile phone users globally [8]. The majority of children and adolescents in 2017 have use of a mobile phone (72% of children aged 0-11 years and 96% of those aged 12-17 years) [9]. Mobile tablet use is also increasing with seven in 10 (71%) children aged 5 to 15 years having access to a tablet at home [10]. Therefore, mobile health (mHealth) offers a particularly powerful and ubiquitous platform for delivering mental health interventions to adolescents. mHealth uses the functions of a mobile device, but most commonly relies on the download of mobile apps to help support health care delivery [11]. More than 15,000 mobile apps for health care were identified in a recent survey with at least 29% designed for mental health [12]. These apps vary in function and may focus on symptom assessment, psychoeducation, promoting engagement with therapy homework (eg, a thought diary or activity schedule), practicing skills learned in therapy, or monitoring symptoms or mood [11].

Advantages of mHealth include constant availability, greater access, equity of mental health resources, immediate support, anonymity, tailored content, lower cost, and increasing service capacity and efficiency [13]. Apps may overcome geographical barriers to treatment and engage traditionally hard-to-reach groups. It has been suggested that technology-based approaches may be particularly suited for children and young people who may be more accepting of technology [14]. Apps may reduce barriers to face-to-face help-seeking, such as the stigma or discomfort about discussing one’s own mental health [15]. Therefore, mental health apps may engage young people who typically would not seek help through traditional routes. Global and national organizations, such as the World Health Organization (WHO), the US Department of Health and Human Services, and the National Health Service (NHS), are generating initiatives for the integration of mHealth in services, including child and adolescent mental health [16,17].

Despite the large number of apps available, the evidence base is scarce, particularly for adolescents. A 2013 review of mobile mental health apps for all ages identified eight papers describing only five apps [18]. Four of the five apps demonstrated significant reductions in depression, stress, and substance use, although a number of issues with the quality of these studies suggest these conclusions needed to be interpreted cautiously. The review also highlighted how research has lagged behind app development. A review of mHealth apps for the most prevalent conditions identified by the WHO identified more than 1536 apps for depression, but only 32 associated published articles [19]. Content analysis of commercially available apps for depression [20] and bipolar disorder [21] demonstrate a concerning trend that downloadable apps may not necessarily reflect evidence-based treatment guidelines. The majority rarely cite source information and often lack privacy policies. This was also evidenced in the now offline NHS App Library, in which only four of the 27 apps included any evidence of patient-reported outcomes to corroborate their effectiveness [22]. As such, the majority of mental health apps available for download are not supported by evidence-based research and may not follow evidence-based treatment guidelines.

Few apps have been specifically developed for children and adolescents, and the benefit of mental health mobile apps for this population is unclear. Two systematic reviews exploring the evidence for digital health interventions (including computerized CBT, mobile phone apps, and wearable technologies) for children and young people with mental health problems in 2014 and 2016 [6,23,24] identified randomized controlled trials (RCTs) for only two apps (Mobiletype and FindMe). Results showed no significant benefits of these apps on depression or autism spectrum disorder symptoms. A scoping review of mHealth interventions for children and young people yielded similar results [25]. Only one app (Mayo Clinic Anxiety Coach) included outcomes using a standardized rating scale, whereas the other two apps identified (SmartCAT and Mobile Mood Diary) had feasibility outcomes, but no efficacy outcomes reported [25].

Although important additions to the literature, the systematic reviews only included RCTs and so did not include feasibility studies providing information on acceptability [6,23,24]. The scoping review was limited to three databases and focused exclusively on studies in which participants had a diagnosed mental health problem [25], therefore excluding any preventive or general mental well-being apps that may exist. This review aims to provide a contemporary appraisal of the available research evidence for the efficacy and acceptability of mobile apps to support the management of mental health in adolescents. A secondary aim was to collate the feedback from mental health professionals and adolescents involved in these studies. This review will focus on mobile phone apps only (as opposed to broad mHealth and eHealth interventions) and will include a wide remit of publication types. Given the increasing number of commercially available apps and the policy drivers toward integrating mHealth into mental health services [16,17], such a review is timely.

## Methods

### Study Identification and Selection

Fifteen electronic databases were searched for relevant publications between January 2008 and July 2016, including APA PsychNet, ACM Digital Library, Cochrane Library, Community Care Inform-Children, EMBASE, Google Scholar, PubMed, Scopus, Social Policy and Practice, and Web of Science. Publication databases of key journals were also searched. These included *Journal of Medical Internet Research*, *Cyberpsychology*, *Behavior and Social Networking*, and *Internet Interventions*. A grey literature search of OpenGrey, Index to Thesis, and ACM Digital Library was also conducted. Words pertaining to mobile apps and devices, mental health problems, and the age of the study population were used in a main search string (see Multimedia Appendix 1 for full search strings by database). Database-specific filters such as human population, English language, and age groups were applied where available. Authors of identified trial protocols were also contacted to determine the current status of these trials and whether any further data were available.

We included abstracts describing mental health apps for mobile devices (mobile phone or tablet) for use by children and adolescents younger than 18 years. Studies with participants older than 18 years were included if the sample included children younger than 18 years. Mental health problems included depression, bipolar disorder, anxiety disorders, self-harm, suicide prevention, conduct disorder, eating disorders and body image issues, schizophrenia, psychosis, and insomnia. To ensure we were capturing current and emerging evidence, we included conference proceedings, theses, case studies, RCTs, uncontrolled feasibility studies, qualitative studies, articles analyzing apps for adolescents available in app stores, and articles detailing app design and development.

We excluded abstracts if (1) the target population was exclusively adult (ie, older than 18 years); (2) the primary purpose of the app was ecological momentary assessment for research purposes as opposed to an intervention; (3) the app was designed for neurodevelopmental disorders (autism spectrum disorders, Asperger syndrome, and attention-deficit/ hyperactivity disorder), for substance use, health behaviors, or medical problems; (4) the study described an Internet-based intervention accessed via a mobile device or an intervention delivered via mobile device functions (text messaging, multimedia messaging, calls, videoconferencing, sending content to Internet interventions); and (5) the paper was a trial protocol, trial registration, systematic or scoping review, or did not provide any extractable outcome or feasibility data.

## Results

### Study Inclusion

**Figure 1:**
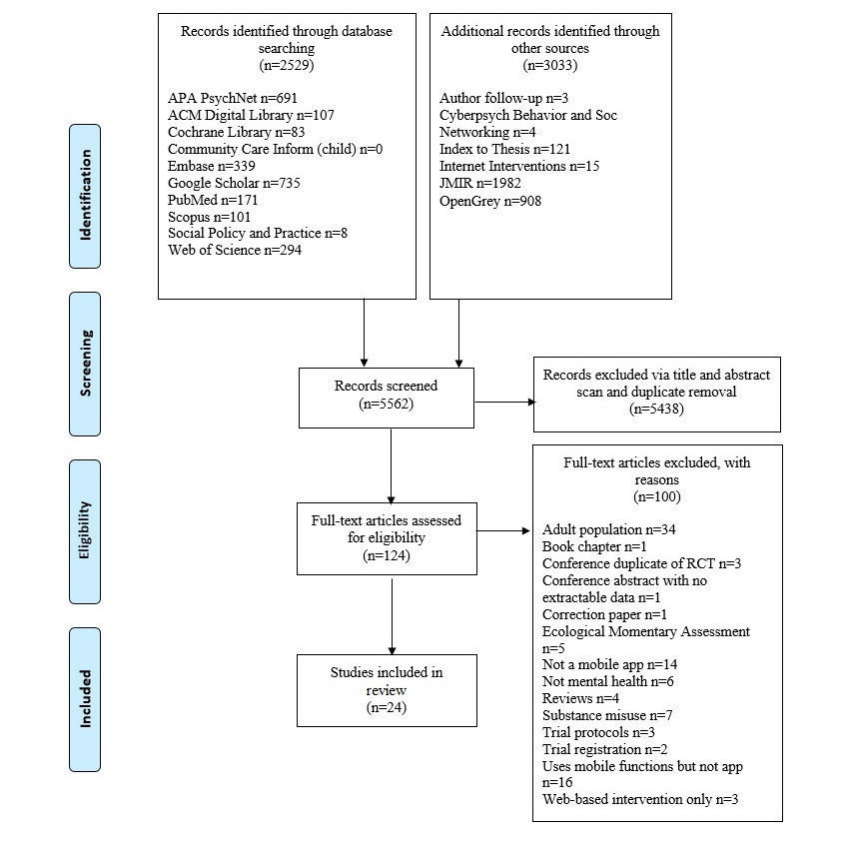
PRISMA flow diagram of results and article selection.

Of the 5562 abstracts initially identified, 5438 were excluded on the basis of title, abstract screening, and duplicate removal. The remaining 124 full-text articles were assessed for eligibility with a further 100 being excluded. A total of 24 full-text articles met the inclusion criteria. Figure 1 is a flow diagram detailing the review process and results at each stage.

### Study Characteristics

The 24 publications included in this review consisted of 12 feasibility studies [26-37], five design and development papers [38-42], and two analyses articles of existing apps in app stores [21,43]. The remaining five reported mental health outcome data [44-48]; of these papers, three reported outcomes from the same RCT [44-46]. Only two studies randomized individuals to trial conditions (Mobiletype RCT [44-46]; Pretty [47]). Publication dates ranged from 2008 to 2016 with a notable increase in publications since 2014. Table 1 reports selected study characteristics.

### Mobile App Characteristics

Table 2 summarizes the 15 apps identified in this review: CopeSmart [26,39], Crisis Care [30], Daybuilder [27], Mayo Clinic Anxiety Coach [42,48], Mobiletype [34,35,44-46], Mobile Mood Diary [28,29], Pretty [47], REACH app [32], Recovery Record [40], Safety Plan app [38], SmartCAT [33], The ACT app [41], and TickiT [37] (two apps had no name [31,36]).

Operating platforms included Android and iOS (n=3 [26,39,40,47]), Android only (n=4 [27,32,33,41]), iOS only (n=2 [37,42,48]), and multiple platforms (n=2 [28,29,34,35, 44-46]), with four being under development or not reporting the operating platform [30,31,36,38]. Note that CopeSmart, Mayo Clinic Anxiety Coach, Mobiletype, and Mobile Mood Diary were associated with multiple studies (Table 2). The primary focus of the apps were prevention and early intervention (n=4 [26,31,32,39,47]), assessment and screening (n=2 [34,35,37,44-46]), adjuncts to face-to-face mental health care (n=5 [28,29,33,36,38,41]), and standalone self-help interventions (n=4 [27,30,40,42,48]). The majority included some form of self-monitoring of symptoms, mood, emotions, behavior, or meals. The Mayo Clinic Anxiety Coach was the only app describing an active “treatment” component (ie, exposure and response prevention) although a further eight provided “coping strategies” and skills practice (eg, meditation, dialectical behavioral therapy [DBT] skills, and CBT techniques).

Two apps were available from Google Play or iTunes at the time of writing: Mayo Clinic Anxiety Coach (iTunes [42,48]) and Recovery Record (Google Play and iTunes [40]). Currently, Recovery Record has not published an evaluation of mental health outcomes but have RCTs registered to take place. As far as can be determined, none of these apps were specifically designed for use with children and young people.

### Analysis Articles

A further six apps, targeted specifically at children or adolescents, were identified in two analysis articles of apps available from Google Play and iTunes [21,43]. The apps were Destructive Issues [43], Teen Depression Connect [43], Teen Hotline [43], Primary School Assessments [21], Preschooler Assessments [21], and Your Child’s Social Health [21]. These analyses concluded that none of these apps have been subject to research evaluation and that the content of some did not reflect best practice guidelines. Further concerns included a lack of privacy policies and lack of resources for immediate help for those who are distressed.

### Study Participants

Ages of those involved in studies ranged from 9 to 30 years with 13 articles including only children and adolescents 18 years or younger [26,28-30,33,34,36-39,42,47,48]. Demographic data including gender were sparsely reported. In total, 473 participants had used a mental health app as part of a feasibility or outcome study [26-28,31-37,44-48]. Of those younger than 18 years (n=316), only a small minority (22/316, 7.0%) had a recognized mental health problem identified by diagnostic interviews or screening questionnaires. In total, 95 adolescents had taken part in studies that evaluated their opinions on mHealth apps or prototypes without using the app itself [28,30,34,38,39]. Only 30 of 95 participants who took part in these studies (32%) had recognized mental health problems.

**Table 1:** Characteristics of publications of mental health mobile apps for preadolescents and adolescents included in review (N=24).

Study	Design^a^	Sample^a^	App
Aguirre et al (2013) [43]	App analysis: mobile apps for suicide prevention from Google Play and iOS	27 apps identified, 3 apps for children and young people	Destructive Issues, Teen Depression, and Teen Hotline
Kauer et al (2012) [44]	Outcome study: RCT of Mobiletype app vs abbreviated Mobiletype app	N=114 (68 intervention; 46 control) aged 14-24; GP-based recruitment	Mobiletype
Kennard et al (2015) [38]	App design: semistructured interviews gaining perspectives on a mobile safety plan for suicide prevention	N=10 teens aged 14-17 hospitalized for suicidality; n=10 parents	Safety Plan App
Kenny et al (2014) [39]	App design: focus groups gaining perspectives on mental health mobile apps and CopeSmart prototype	N=34, aged 15-16, school-based sample	CopeSmart
Kenny et al (2015) [26]	Feasibility: CopeSmart used to rate mood for 1 week	N=43, aged 15-17, school-based sample	CopeSmart
Løventoft et al (2012) [27]	Feasibility: describes design workshops and 4-week pilot trial	N=6 (aged 17-24); used psychotropic medication within last 2 years; community recruited	Daybuilder
Matthews & Doherty (2011) [28]	Feasibility: comprises 3 studies (1) initial design consultations, (2) nonclinical feasibility, (3) feasibility with clinical population	(1) n=6, (2) n=73 (21 app, 51 paper diary), (3) n=9 children seeing a therapist for a range of mental health problems	Mobile Mood Diary
Matthews et al (2008) [29]	Feasibility: app or paper-based mood charting; instructed to complete one mood entry every day for 2 weeks	N=73 (21 app, 51 paper diary), aged 13-17 years; school-recruited sample	Mobile Mood Diary
McManama et al (2016) [30]	Feasibility: pilot testing of prototype of app for suicide prevention following acute care discharge; think-aloud protocol	N=20 aged 13-18, history of suicidal thoughts and n=20 parents; outpatient psychiatry dept-recruited sample	Crisis Care
Nicolas et al (2015) [21]	App analysis: mobile apps (English language) for bipolar disorder from the Australian Google Play and iOS in 2014	82 apps identified 3 specifically for children and young people	Primary School Assessments, Preschooler Assessments & Your Child’s Social Health
Niendam et al (2015) [31]	Feasibility: 4-month trial collecting medication adherence and clinical data using mobile phone app	N=36, aged 14-30; Early Psychosis participants recruited from early intervention programs	No name
Patwardhan et al (2015) [32]	Feasibility: pilot of REACH app; 30 minutes of app usage with researcher	N=22 (mean age=9.67 years); school-based recruitment	The REACH app
Pramana et al (2014) [33]	Feasibility: used for 8-16 alongside face-to-face CBT for anxiety	N=9 (aged 9-14), receiving face-to-face CBT for diagnosed anxiety disorder	SmartCat
Reid et al (2009) [34]	Feasibility: focus group and 1-week trial of Mobiletype; text prompt to complete diary 4 times a day	N=29 (n=11 in focus group, n=18 in study), aged 14-17; school-based recruitment	Mobiletype
Reid et al (2011) [45]	Outcome study: RCT of Mobiletype app vs abbreviated Mobiletype app	N=114 (68 intervention; 46 control) aged 14-24; GP-based recruitment	Mobiletype
Reid et al (2012) [35]	Feasibility: youth asked to self-monitor with app at least once a day for 2-4 weeks until next medical review	N=47 (aged 14-19), recruited from health clinic by pediatrician	Mobiletype
Reid et al (2013) [46]	Outcome study: RCT of Mobiletype app vs abbreviated Mobiletype app	N=114 (68 intervention; 46 control) aged 14-24; GP-based recruitment	Mobiletype
Scotti (2014) [36]	Feasibility: school-based DBT skills group + mobile or online tracking of skills usage	N=7 (aged 13-18), 2 of which used the app; had eating disorder or body image concerns; school-based recruitment	No name
Tregarthen et al (2015) [40]	App design: app made available to download and user information recorded	Ages ranged from 13-77 years	Recovery Record
Veldhuis (2014) [47]	Outcome study: app for body image or neutral app used in laboratory for 30 minutes	N=206 (aged 12-18); school-based recruitment	Pretty
Verstappen et al (2014) [41]	App design: development of ACT app for youth with depression learning ACT	Mentions “research clients” as a group of 15 “youth” undertaking 3-month ACT program at health center	The ACT app
Whitehouse et al (2013) [37]	Feasibility: piloting use of psychosocial screening app in a medical hospital setting	N=80 medical patients aged 12-18; recruited in medical clinics before appointments	TickIT
Whiteside et al (2014) [48]	Outcome study: case studies of two children with OCD using Mayo Clinic Anxiety Coach alongside face-to-face therapy for 3 months	N=2 (10 and 16 years) both diagnosed with OCD; mental health clinic-recruited	Mayo Clinic Anxiety Coach
Whiteside et al (2016) [42]	App design: user data from downloaders of Mayo Clinic Anxiety Coach	User data: children and adolescents 5-17 downloaded Mayo Clinic Anxiety Coach (likely with parents)	Mayo Clinic Anxiety Coach

^a^ ACT: acceptance and commitment therapy; app analysis: article on app analysis; app design: article on app design and development; DBT: dialectical behavioral therapy; GP: general practitioner; OCD: obsessive-compulsive disorder; outcome study: study reporting mental health outcomes.

### Mental Health Outcomes: Efficacy

As evident in Table 2, the included apps targeted a range of mental health areas. However, only five articles reported any mental health outcome data. These articles evaluated three apps targeting depression, stress, anxiety (Mobiletype [44-46]), body image and self-esteem (Pretty [47]), and obsessive-compulsive disorder (OCD; Mayo Clinic Anxiety Coach [48]). None of the other targeted areas had any outcome data associated with them.

#### Depression, Stress, and Anxiety

To date, Mobiletype is the only mobile app to have undergone a RCT [44-46]. Participants (N=118, aged 14-24 years) with emotional or mental health issues were recruited from general practitioner practices and randomly assigned to a full or an abbreviated version of Mobiletype (ie, no mental health self-monitoring). There were no significant differences between groups at posttest or follow-up (6 weeks) on depression (*d=* 0.09, *P*=.69), anxiety (*d=* 0.07, *P*=.76), or stress (*d=* 0.22, *P*=.32) as measured by the Depression, Anxiety and Stress Scale (DASS). Significant total group mean decreases on the DASS over time indicated significant reductions in depression, anxiety, and stress scores at follow-up regardless of group [44-46].

#### Body Image and Self-Esteem

Veldhuis [47] reported a laboratory-based, randomized trial of a mobile phone app (Pretty) to improve body image in a community sample of 206 adolescent girls (age 12-18 years, mean 13.88, SD 1.34 years). Participants were randomized to use Pretty or a comparison app for 30 minutes. Both apps presented pictures of models. Pretty asked users to rate the weight status of each model, whereas the comparison app asked neutral questions about a famous Dutch singing duo. There were no significant differences between apps on measures of self-esteem or body satisfaction postapp exposure (*t*_1_=0.02, *P*=.90 and *t*_1_=0.54, *P*=.46, respectively). Neither app improved body satisfaction; however, significant improvements in self-esteem postapp use were revealed, regardless of which app was used (*t*_20_=–4.26, *P*=.001).

#### Obsessive-Compulsive Disorder

Whiteside [48] presented two case studies (ages 10 and 16 years) illustrating the treatment of pediatric OCD augmented with the Mayo Clinic Anxiety Coach App. Posttreatment assessments were at 4 and 3 months, respectively. Although symptoms on the Children’s Yale-Brown Obsessive-Compulsive Scale were reduced, one child still met diagnostic criteria for OCD [48].

### Feasibility and Acceptability

Feasibility outcomes of app usage and acceptability were extracted from studies assessing the following apps: CopeSmart [26,39], Crisis Care [30], Daybuilder [27], Mobiletype [34,35,44], Mobile Mood Diary [28,29], SmartCAT [33], REACH app [32], and TickiT [37]. Qualitative feedback from adolescents and therapists [26,28,29,39] was also reported.

#### App Usage

In the Mobiletype RCT [44,45], app users (N=68) were instructed to use the app at least twice a day for a minimum of 2 weeks. App use was good with participants completing a mean 3.3 (SD 1.4, range 1-8) Mobiletype entries each day and average app usage of mean 14.6 (SD 6.3, range 1-34) days. In a feasibility trial of CopeSmart [26], a nonclinical sample of adolescents (N=43) used the app for a mean 4.0 of 7.0 (SD 1.8) days. The “Rate My Mood” section was most frequently used (mean 3.5, SD 1.0 days), whereas use of the “Coping Tips” and “Resources” sections were low (mean 1.5, SD 1.0 days and mean 0.9, SD 1.0 days, respectively).

**Table 2:** Characteristics of mental health mobile apps for preadolescents and adolescents included in review (N=15).

App name	Description^a^	Main features^a^	OS^b^	Available to download^c^	Area targeted^d^
CopeSmart [26,39]	App to foster positive mental health in children and young people	Self-monitoring of mood, mood diary, coping tips, and contact details of mental health support services	Android & iOS	NA	Mental well-being
Crisis Care [30]	App for suicide prevention in children and young people to be downloaded on discharge from acute care	Coping skills (relaxation, behavioral activation, positive affect) and contact details of suicide hotline and adults they trust	Prototype /NR	NA	Suicide prevention
Daybuilder [27]	A “life management app” for people with depression	Symptom assessment, mood, appetite, and sleep self-monitoring, functions to let the user create events and reminders for what to do to prepare for that event, medication management	Android	NA	Depression
Mayo Clinic Anxiety Coach [42,48]	A self-help tool delivering CBT for a range of anxiety disorders	Self-monitoring, symptom assessment, psychoeducation, and treatment based on exposure therapy	iOS	Yes	OCD
Mobiletype [34,35,44-46]	A “mental health assessment and management app” for children and young people	Self-monitoring tool; prompts users 4 times a day to record mood, stressful events, alcohol use, cannabis use, quality and quantity of sleep, quantity and type of exercise, and diet	Cross- platform	NA	Mental health
Mobile Mood Diary [28,29]	App for children and young people in therapy to chart their mood	Self-monitoring of mood, sleep, and energy and a free text diary entry; no password protection or reminders	Cross- platform	NA	Mental health
Pretty [47]	Gamified app to prevent body image issues in children and young people	App is a series of pictures of models of various sizes and questions asking the user to rate each model’s weight status to be either “extremely thin,” “thin,” “normal,” “big,” or “extremely big;” user gets feedback on whether their response was correct	Android & iOS	NA	Body image
REACH app [32]	App for anxiety prevention and early intervention in children and young people	Self-monitoring, resources, coping strategies, and CBT skills training	Android	NA	Anxiety
Recovery Record [40]	A CBT-based app for eating disorders self-monitoring	Self-monitoring of meals and symptoms, goal setting, coping tactics, meal plans, rewards and affirmations, social support, summative feedback	Android & iOS	Yes	Eating disorders
Safety Plan app [38]	Proposed app to support children and young people transitioning from inpatient to outpatient care	Intended to provide mobile access to pre-agreed safety plan for use in times of crisis and suicidal ideation	Prototype /NR	NA	Suicide prevention
SmartCAT [33]	App for children and young people with anxiety alongside brief CBT sessions	Skills coach, reward bank, media library, notifications, and secure messaging portal for use with therapist	Android	NA	Anxiety
The ACT app [41]	App for children and young people with depression attending therapy	Self-monitoring and symptom assessment, skills training, goal setting; based on acceptance and commitment therapy.	Android	NA	Depression
TickiT [37]	App-based psychosocial screening tool developed for children and young people attending hospital	Patients enter data in waiting room and the tool records response data, generating a report and alerts for clinicians, shifting clinical focus of the meeting	iOS	NA	Depression (screening)
No name [31]	App for recording medication adherence and symptoms in early psychosis care	Self-monitoring and symptom assessment; designed with daily and weekly surveys assessing symptoms, mood, medication adherence, and social contact	NR	NA	Early psychosis (medication adherence)
No name [36]	App for recording behaviors and skills practice, adjunct to group DBT	Self-monitoring and tracking of DBT skills and ED behaviors via mobile app or online	NR	NA	Eating disorders

^a^ CBT: cognitive behavioral therapy; DBT: dialectical behavioral therapy; ED: eating disorders.

^b^ Cross-platform: article reports as JavaME app (Mobile Mood Diary) or “multiple models and firmware” (Mobiletype); NR: not reported; OS=operating system.

^c^ NA: not available to download from Google Play, iTunes App Store, or Microsoft app store.

^d^ Mental health: range of unspecified mental health problems.

In a feasibility trial of Mobile Mood Diary [28,29], a nonclinical sample of school children (N=73, aged 13-17 years) were asked to record at least one mood each day for 2 weeks. App users demonstrated significantly higher levels of compliance (entries: mean 8.12) compared to a control group who mood-charted with a pen and paper (mean 5.44). In a small pilot study [28] with a clinical population of children (N=9; age: mean 13.78, SD 2.63 years) attending therapy, mood diary adherence was 65% on average. All participants used Mobile Mood Diary for a minimum of two sessions and 8 of 9 (89%) used it for longer. In a SmartCAT feasibility trial [33], clinically anxious youth (N=9, aged 9-14 years) demonstrated good compliance, completing a mean 5.36 (SD 1.95) entries of 6.48 requests (5.36/6.48, 83% adherence rate) between each session. There was limited data about the longer-term use of apps. SmartCAT was highly utilized during week 1, but leveled off over time and almost halving by week 7 [33]. Similarly, in a feasibility trial of Mobiletype [35], participants (N *=* 47, age 14-24 years) completed 91% (47/51) of the Mobiletype entries every day in week 1, dropping to 58% (17/29) in week 4.

#### App Acceptability

Sample sizes were small, but overall app acceptability was good. The majority of CopeSmart users in the feasibility trial [26] found the app easy to use (40/43, 93%), 30 of 43 (70%) would use it in the future, 32 of 43 (74%) felt other young people would use it, and 30 of 43 (70%) would recommend it to a friend. Similarly, SmartCAT feasibility study participants [33] rated the app as highly usable (mean 1.7 on a scale of 0-7 with 1 indicating easy to use). All users reported being satisfied with SmartCAT and would recommend to others. The REACH app [32] (N *=* 22, age mean 9.67 years) was rated highly on ease of use, quality of support information, ease of learning, and system satisfaction with an overall mean usability score of 35.69 (SD 19.84) out of a possible score of 40. Participants who had no knowledge of the Android operating system rated the app worse. Users (N=21) of Mobile Mood Diary [29] also found it easy to use (mean 1.63, SD 0.76, where 1=very easy and 5=very difficult). Furthermore, 20 of 21 (95%) felt they had sufficient privacy and felt more privacy recording moods via the app compared to paper-based charting [29]. A feasibility study of TickiT (N=78, age range 12-18 years) demonstrated the app was easy to understand (72/78, 92%), easy to use (72/78, 92%), and efficient (63/79, 80%), with a completion rate of less than 10 minutes [37]. For participants in the Mobiletype feasibility study, 21 of 22 (95%) reported the feedback information reflected their actual experiences, was accurate (95%, 20/21), was helpful to them (71%, 17/24), and aided their doctor to understand them better (82%, 18/22) [35]. Usability of Crisis Care in a pilot study [30] was judged to be good (N=20). Mean scores on usability, utility in crisis, and content satisfaction ranged from 2 to 5 (5 being maximum score on subscales of System Usability Scale).

#### Adolescent Perspectives

Feedback from a focus group of nonclinical adolescents (N=34, age 15-16 years) highlighted the importance of apps being discrete and easy to conceal in order to avoid the stigma associated with mental health problems [39]. Privacy concerns were also highlighted by three participants from a clinical sample who declined to use Mobile Mood Diary in a pilot study because the title would be visible on their phone (eg, “one 16-year-old would not install the diary because her friends sometimes use her phone and she is afraid they will see an application named ‘mood diary’” p 2954 [28]). Others report mobile apps offer increased privacy and discretion for activities, such as mood charting. A participant in the Mobile Mood Diary feasibility trial commented, “You can conceal more easily so there is more privacy” (p 123 [29]). On a practical level, adolescents would like apps to have password protection and to allow control over privacy settings [28,29,39]. Other feedback highlighted that apps should also be engaging, interactive, provide concise information, be esthetically attractive, allow for personalization, and provide reminders to use [26,28,29].

#### Therapist Perspectives

A survey [28] of therapist attitudes to mobile technology (N=28) revealed concerns about privacy and security. The “danger of someone else accessing confidential information” was a substantial therapist concern. Other concerns included increased responsibility, increased workloads, costs of implementation, need for training, setting clear boundaries between sessions, and a worry that clients would expect the therapist to continuously monitor their mood data [28]. Therapists (n=3) who used Mobile Mood Diary in a pilot study reported the app and printouts helped engage patients in therapeutic tasks, facilitated a less threatening disclosure of information, and broke down barriers in sessions. Mobile mood charting was perceived to be better than paper-based charting and printouts were useful for discussing clinical cases and saved therapist time inputting into a computer [28]. Lack of technical confidence was reported to be the greatest barrier to uptake of Mobile Mood Diary. Some therapists were incentivized to use the app when they saw others successfully using it.

## Discussion

### Principal Results and Comparisons With Previous Work

The aim of this review was to systematically examine the literature on mobile apps for mental health in children and young people. Our review identified 24 papers describing 15 apps or prototypes, two of which were available to download from Google Play or iTunes [40,42,48]. We identified only two small RCTs [44-47], one of which was a laboratory-based experimental study [47], and both failed to demonstrate a significant effect on their intended outcomes (depression or body image). Therefore, we conclude that currently there is no evidence to support the effectiveness of apps designed for adolescents with mental health problems. Because we did not identify any study participants younger than 9 years, there is no evidence to support the effectiveness of apps designed for children with mental health problems either.

Our conclusion is consistent with previous reviews and highlights that the evidence base has barely increased over the past 4 years [18,23-25]. The lack of empirical studies contrasts starkly with the commercial development of mobile apps. From October 2013 to June 2016, the number of apps available to download from the app store doubled to 2 million [49], of which 1.98% (39,600) were classed as medical. Given the significant increase in the availability of mobile apps, the lack of evidence to support their safety or effectiveness with vulnerable populations is concerning.

Although the evidence base is currently lacking, this does not rule out the fact that well-designed, adequately tested, evidenced-based mobile apps could be effective. The evidence base for the clinical effectiveness of mobile apps in adult mental health is slowly emerging [18,50-52]. Our review suggests that the comparative literature for children and adolescents is significantly lagging, a trend also noted within the literature on other forms of eHealth, such as computerized CBT [6,7].

In terms of acceptability, it has been suggested that apps and eHealth in general are particularly suited for adolescents who are familiar with and regular users of technology [23]. We identified 12 small pilot feasibility trials [26-37] that suggested, in the short term, adolescents may be favorably disposed to this form of delivery. Acceptability was generally positive with ratings of ease of use, satisfaction, and usability rated average to high [26,29,32,33,35,37]. The privacy and discretion afforded by well-designed apps were of importance to young people [28,29,39]. However, many participants were healthy, nonreferred adolescents and less is known about whether those with mental health problems would have similar views. It is important to also note that although adolescents may have positive attitudes toward mHealth, it does not necessarily mean they would prefer it over a face-to-face intervention [24]. It is also important to consider whether the affinity that people have for their mobile phones and the trust and expectations placed in them positively influence clinical outcomes and user satisfaction [53]. This “digital placebo effect” may account for why some people continue to download and use mobile apps for mental health even though the evidence base is largely absent [53]. Nonetheless, our findings support previous conclusions and suggest that apps may provide an acceptable way of supporting mental health interventions for some adolescents [18,54].

Therapist perspectives on mobile apps were mixed, with concerns relating to patient security, increased responsibility and workloads, and the need to set clear boundaries between sessions [28]. These are different concerns to those surrounding the use of other forms of eHealth, such as computerized CBT, in which clinicians were concerned about the effectiveness of computerized CBT with more severe mental health problems and the lack of a therapeutic relationship [55]. This may reflect differences in the purpose of these interventions (ie, computerized CBT being a therapeutic intervention compared to apps that are an adjunct to therapy). Interestingly, therapists who used the app Mobile Mood Diary in clinical practice reported benefits such as facilitation of client engagement [28]. Lack of technical confidence was the most common barrier to implementation. This lack of technical confidence may be addressed by improving the user-friendliness of the app, either by codesigning apps with therapists or providing training for therapists.

App usage, where reported, was moderate and adherence ranged from 65% to 83%, which is comparable to those seen in Internet interventions for depression and anxiety [56]. There was a suggestion that self-monitoring of mood via apps promoted higher adherence compared to paper self-monitoring [33,35]. Information on longer-term usage is scarce, but the included studies suggest app usage begins high and declines over time [33,35]. This “law of attrition” [57] is also a common challenge for computerized CBT and eHealth interventions [57]. As with these other technology-based interventions, using mHealth apps with support from a therapist offers one strategy for increasing longer-term engagement [24,58]. Indeed, the SmartCAT app used in conjunction with face-to-face support demonstrated an 83% completion rate [33], similar to completion rates demonstrated in face-to-face CBT (84%) and guided Internet CBT (81%) [59]. Making mHealth apps inherently more engaging by design is another strategy for increasing longer-term engagement. One promising proposition is the use of serious gaming, gamification principles, telepresence, and persuasive technology in eHealth (and by extension mHealth) design [58,60]. The evidence base for the benefits of these principles as applied to mHealth and eHealth is currently in its infancy, however, and is a burgeoning area of research [60].

This review highlights several methodological concerns about the quality of the research evidence for mental health mobile apps, especially those for adolescents. Sample sizes tend to be small and reporting of demographic data such as gender and age inadequate, particularly in pilot feasibility studies. Few participants have an identified mental health problem and, as such, little is known about the acceptability and use of apps with clinical groups. As far as can be determined, the youngest participant in these studies was 9-years-old, meaning there is no research evidence for the use of mobile apps in children younger than this age. Where reported, symptoms tended to be mild to moderate in severity and, as such, the appropriateness of mobile apps for complex or more severe problems is unknown. Studies tend to be short in duration and there is sparse information on whether positive gains from using mobile apps are maintained. Finally, none of the apps in this review have been evaluated using a suitable RCT comparing a mobile app to an adequate control group. Future research should address these methodological concerns. Given the beneficial role that parent participation and engagement can have in adolescent mental health treatment [61], future research may also want to consider the role of parents/guardians in supporting adolescents using apps for mental health.

Our review has focused on the academic literature and of the apps identified, two of which were available to download. This contrasts starkly with the large number available from commercial sites and raises questions about the safety, quality, and efficacy of those that are available [11,22,62]. Content analyses [21,43] of six apps for children and adolescents available to download highlighted that none have been subject to any research evaluation. The authors also noted that the apps did not reflect best practice guidelines and lacked privacy policies [21,43]. Some of these apps claim to address a worrying number and type of complex problems, including “child abuse,” “daughter’s abusive relationship,” and “teen suicide, depression, and stress.” Ineffective or detrimental apps are a significant concern and incur costs to patient safety and care [11]. Therefore, our review adds to calls for better regulatory oversight to ensure app quality and safety [11,18,22,62].

### Limitations

There are several limitations of this review. Firstly, the number of studies was small with generally limited sample sizes. Conclusions that mobile apps are acceptable for youth are therefore tentative. Secondly, the qualitative feedback is based on a small number of young people and therapists and generalizing their views to a wider population should be exercised with caution. This feedback is nevertheless informative and highlights the importance of involving young people in app design. Thirdly, we aimed to reduce publication bias, and although our inclusion criteria were broad, our search was limited to English-language papers. Fourthly, despite aiming for a precise overview of the literature on mobile apps for children and adolescents, a number of publications included adults. The majority of publications utilized teenage and young adult populations with only one study including a participant aged 9 years. As such, our results are limited to preadolescents and adolescents, rather than children. All the articles included in this review originated from work in North America, Northern Europe, and Australia; therefore, these results are limited to the experiences of adolescents in high-income countries. mHealth holds great promise for widening access to mental health treatment in low to upper-middle income countries where the challenges of meeting mental health needs are considerable [63]. This potential will not be realized unless future research is conducted in these contexts.

### Conclusion

There is an urgent need for methodologically robust, adequately powered research evaluating the safety, efficacy, and effectiveness of mental health apps for children and young people with mental health problems. Well-designed RCTs with adequate power and control groups are needed to demonstrate whether mobile apps for mental health have any clinical benefit for children and young people. Because the development of apps is vastly outpacing the development of the evidence base, future research should also utilize quicker, good-quality designs [64]. This may require the inclusion of adolescents and therapists in the app design and development process to ensure apps are fit for purpose and user-centered [58], as well as continuous evaluation of evolving interventions [64]. At present, there is insufficient evidence to suggest that any mobile app for mental health can be used effectively with children and young people. Clinicians should be cautious about recommending mobile apps until there is sufficient evidence to support their safety and efficacy.

## Appendix

Multimedia Appendix 1 List of databases and search strings used for systematic review.

## Abbreviations

CBT: cognitive behavioral therapy
DBT: dialectical behavioral therapy
DASS: Depression, Anxiety and Stress Scale
mHealth: mobile health
NHS: National Health Service
RCT: randomized controlled trial
WHO: World Health Organization
