# Supplementary material for: Mental Health Mobile Apps for Preadolescents and Adolescents: A Systematic Review
Source: J Med Internet Res. 2017 May 25;19(5):e176. doi: 10.2196/jmir.7332 (PMC5465380; doi:10.2196/jmir.7332)
Supplement: Multimedia Appendix 1 [file jmir_v19i5e176_app1.pdf]

Multimedia Appendix 1

List of databases and search strings used for systematic review

APA PsychNET

for Any Field : " mobile health" OR " mobile device" OR ((mobile OR smart OR tablet) NEAR/5 (app* OR application* OR phone*)) OR " mobile app" OR " smartphone" OR " mobile phone" AND Any Field : " depress*" OR " self – harm" OR suicid* OR anxi* OR " PTSD" OR " social anxi*" OR " separation anxi*" OR phobia OR " generalised anxiety disorder" OR " OCD" OR " conduct disorder" OR " eating disorder" OR anorexi* OR bulimi* OR " binge eating" OR " body image" OR " mental health*" OR Schizophren* OR " bipolar affective disorder" OR psychos* OR " insomnia*" OR stress* AND Any Field : child* OR teenage* OR adolescen* OR " young per*" OR youth* OR " young adult*" AND Age Group : Childhood (birth to 12 yrs) OR Preschool Age (2 to 5 yrs) OR School Age (6 to 12 yrs) OR Adolescence (13 to 17 yrs) OR Young Adulthood (18 to 29 yrs) AND Population Group : Human AND Year : 2008 To 2016

ACM Digital Library

Searched for “mHealth*” OR “m-health*” OR “mobile health” OR “mobile device” OR “mobile app” OR “smartphone” OR “mobile phone” AND (“depress*” OR “self – harm” OR suicid* OR anxi* OR “PTSD” OR “social anxi*” OR “separation anxi*”OR phobia OR “generalised anxiety disorder” OR “OCD” OR “conduct disorder” OR “eating disorder” OR anorexi*OR bulimi* OR “binge eating” OR “body image” OR “mental health*” OR Schizophren* OR “bipolar affective disorder” OR psychos* OR “insomnia*” OR stress*) AND (child* OR teenage* OR adolescen* OR “young per*” OR youth* OR "young adult*") [new search] [edit/save query] [advanced search]

Searched The ACM Full-Text Collection

Refinements Published since:2008

Cochrane Library

'"mHealth*" OR "m-health*" OR "mobile health" OR "mobile device" OR ((mobile OR smart OR tablet) NEAR/5 (app* OR application* OR phone*)) OR "mobile app" OR "smartphone" OR "mobile phone" in Title, Abstract, Keywords and "depress*" OR "self – harm" OR suicid* OR anxi* OR "PTSD" OR "social anxi*" OR "separation anxi*"OR phobia OR "generalised anxiety disorder" OR "OCD" OR "conduct disorder" OR "eating disorder" OR anorexi*OR bulimi* OR "binge eating" OR "body image" OR "mental health*" OR Schizophren* OR "bipolar affective disorder" OR psychos* OR "insomnia*" OR stress* in Title, Abstract, Keywords and child* OR teenage* OR adolescen* OR "young per*" OR youth* OR "young adult*" in Title, Abstract, Keywords , Publication Year from 2008 to 2016 in Cochrane Reviews'

'"mHealth*" OR "m-health*" OR "mobile health" OR "mobile device" OR ((mobile OR smart OR tablet) NEAR/5 (app* OR application* OR phone*)) OR "mobile app" OR "smartphone" OR "mobile phone" in Title, Abstract, Keywords and "depress*" OR "self – harm" OR suicid* OR anxi* OR "PTSD"

OR "social anxi*" OR "separation anxi*"OR phobia OR "generalised anxiety disorder" OR "OCD" OR "conduct disorder" OR "eating disorder" OR anorexi*OR bulimi* OR "binge eating" OR "body image" OR "mental health*" OR Schizophren* OR "bipolar affective disorder" OR psychos* OR "insomnia*" OR stress* in Title, Abstract, Keywords and child* OR teenage* OR adolescen* OR "young per*" OR youth* OR "young adult*" in Title, Abstract, Keywords , Publication Year from 2008 to 2016 in Trials'

Community Care Inform (Children) 0 results

Home » Mental health » Search results for "(('mhealth*' OR 'm-health*' OR 'mobile health' OR 'mobile device' OR 'smartphone' OR 'mobile app' OR 'mobile phone') AND ('depress*' OR 'self – harm' OR 'suicid*' OR 'anxi*' OR 'ptsd' OR 'social anxi*' OR 'separation anxi*' OR 'phobia' OR 'generalised anxiety disorder' OR 'ocd' OR 'conduct disorder' OR 'eating disorder' OR 'anorexi*' OR 'bulimi*' OR 'binge eating' OR 'body image' OR 'mental health*' OR 'schizophren*' OR 'bipolar affective disorder' OR 'psychos*' OR 'insomnia*' OR stress*) AND ('child*' OR 'teenage*' OR 'adolescent*' OR 'young per*' OR 'youth*' OR 'young adult*'))"

Embase

'mhealth*' OR 'm-health*' OR 'mobile health' OR 'mobile device' OR 'mobile app'/exp OR 'mobile app' OR 'smartphone'/exp OR 'smartphone' OR 'mobile phone'/exp OR 'mobile phone' AND ('depress*' OR 'self – harm' OR 'suicid*' OR 'anxi*' OR 'ptsd'/exp OR 'ptsd' OR 'social anxi*' OR 'separation anxi*' OR 'phobia'/exp OR 'phobia' OR 'generalised anxiety disorder'/exp OR 'generalised anxiety disorder' OR 'ocd' OR 'conduct disorder'/exp OR 'conduct disorder' OR 'eating disorder'/exp OR 'eating disorder' OR 'anorexi*' OR 'bulimi*' OR 'binge eating' OR 'body image'/exp OR 'body image' OR 'mental health*' OR 'schizophren*' OR 'bipolar affective disorder'/exp OR 'bipolar affective disorder' OR 'psychos*' OR 'insomnia*' OR stress*) AND ('child*' OR 'teenage*' OR 'adolescent*' OR 'young per*' OR 'youth*' OR 'young adult*') AND [english]/lim AND ([child]/lim OR [preschool]/lim OR [school]/lim OR [adolescent]/lim OR [young adult]/lim) AND [humans]/lim AND [2008-2016]/py

Index to Thesis (ProQuest)

("mHealth*" OR "m-health*" OR "mobile health" OR "mobile device" OR (mobile OR smart OR tablet) NEAR/5 (app* OR application* OR phone*) OR "mobile app" OR "smartphone" OR "mobile phone") AND ("depress*" OR "self – harm" "suicid*" "anxi*" "PTSD" "social anxi*" "separation anxi*" "phobia" "generalised anxiety disorder" "OCD" "conduct disorder" "eating disorder" "anorexi" "bulimi*" "binge eating" "body image" "mental health*" "schizophren*" "bipolar affective disorder" "psychos" "insomnia*" "stress") AND ("child*" OR "teenage*" "adolescen*" "young per*" "youth*" "young adult*") Filters activated: Publication date: 2008 - 2016 Subject: Children & youth OR mental health OR teenagers OR adolescent OR smartphones OR young adult OR child OR mental disorders OR mental depression

PubMed

((("mHealth*" OR "m-health*" OR "mobile health" OR "mobile device" OR ((mobile OR smart OR tablet) near/5 (app* OR application* OR phone*)) OR "mobile app" OR "smartphone" OR "mobile

phone")) AND ("depress*" OR "self - harm" OR suicid* OR anxi* OR "PTSD" OR "social anxi*" OR "separation anxi* " OR phobia OR "generalised anxiety disorder" OR "OCD" OR "conduct disorder" OR "eating disorder" OR anorexia *or bulimi* OR "binge eating" OR "body image" OR "mental health*" OR Schizophren* OR "bipolar affective disorder" OR psychos* OR "insomnia*" OR stress*)) AND (child* OR teenage* OR adolescen* OR "young per*" OR youth* OR "young adult*") Filters activated: Publication date from 2008/01/01 to 2016/12/31, Humans, English, Child: birth-18 years, Adult: 19+ years

Scopus

(TITLE-ABS-KEY ("mHealth*" OR "m-health*" OR "mobile health" OR "mobile device" OR "smartphone" OR "mobile app" OR "mobile phone") AND TITLE-ABS-KEY ("depress*" OR "self -- harm" OR "suicid*" OR "anxi*" OR "PTSD" OR "social anxi*" OR "separation anxi*" OR "phobia" OR "generalised anxiety disorder" OR "OCD" OR "conduct disorder" OR "eating disorder" OR "anorexi*" OR "bulimi*" OR "binge eating") OR TITLE-ABS-KEY ("body image" OR "mental health*" OR "schizophren*" OR "bipolar affective disorder" OR "psychos*" OR "insomnia*"OR "stress*") AND TITLE-ABS-KEY ("child*" OR "teenage*" OR "adolescen*" OR "young per*" OR "youth*" OR "young adult*")) AND PUBYEAR > 2007 AND (LIMIT-TO (LANGUAGE , "English"))

Social Policy and Practice (Ovid search)

(("mHealth*" or "m-health*" or "mobile health" or "mobile device" or "smartphone" or "mobile app" or "mobile phone") and ("depress*" or "self – harm" or "suicid*" or "anxi*" or "PTSD" or "social anxi*" or "separation anxi*" or "phobia" or "generalised anxiety disorder" or "OCD" or "conduct disorder" or "eating disorder" or "anorexi*" or "bulimi*" or "binge eating" or "body image" or "mental health*" or "Schizophren*" or "bipolar affective disorder" or "psychos*" or "insomnia*or stress*") and ("child*" or "teenage*" or "adolescen*" or "young per*" or "youth*" or "young adult*")) limit 1 to yr="2008 -Current"

Web of Science

(“mHealth*” OR “m-health*” OR “mobile health” OR “mobile device” OR (mobile OR smart OR tablet) NEAR/5 (app* OR application* OR phone*) OR “mobile app” OR “smartphone” OR “mobile phone”) AND TOPIC: (“depress*” OR “self – harm” OR suicid* OR anxi* OR “PTSD” OR “social anxi*” OR “separation anxi*”OR phobia OR “generalised anxiety disorder” OR “OCD” OR “conduct disorder” OR “eating disorder” OR anorexi*OR bulimi* OR “binge eating” OR “body image” OR “mental health*” OR Schizophren* OR “bipolar affective disorder” OR psychos* OR “insomnia*” OR stress*) AND TOPIC: (child* OR teenage* OR adolescen* OR “young per*” OR youth* OR "young adult*") Timespan: 2008-2016. Indexes: SCI-EXPANDED, SSCI, A&HCI, CPCI-S, CPCI-SSH, BKCI-S, BKCI-SSH, ESCI.

Google Scholar

'mhealth*' OR 'm-health*' OR 'mobile app'' AND 'depressed*' OR 'self – harm' OR 'suicide*' OR 'anxi*' OR 'conduct disorder' OR 'eating disorder' OR 'mental health*' OR 'schizophren*' OR 'bipolar' OR 'psychos*' AND 'child*' OR 'teenage*' OR 'adolescent* LIMIT DATE RANGE 2008 - 2016

OpenGrey

"mHealth*" OR "m-health*" OR "mobile health" OR "mobile device" OR ((mobile OR smart OR tablet) NEAR/5 (app* OR application* OR phone*)) OR "mobile app" OR "smartphone" OR "mobile phone")) AND ("depress*" OR "self – harm" OR suicid* OR anxi* OR "PTSD" OR "social anxi*" OR "separation anxi* "OR phobia OR "generalised anxiety disorder" OR "OCD" OR "conduct disorder" OR "eating disorder" OR anorexi*OR bulimi* OR "binge eating" OR "body image" OR "mental health*" OR Schizophren* OR "bipolar affective disorder" OR psychos* OR "insomnia*" OR stress*) AND (child* OR teenage* OR adolescen* OR "young per*" OR youth* OR "young adult*")

Journal of Medical Internet Research (all publications)

"mHealth*" OR "m-health*" OR "mobile health" OR "mobile device" OR "mobile app" OR "smartphone" OR "mobile phone" AND child* OR teenage* OR adolescen* OR "young per*" OR youth* OR "young adult*" AND mental health.

Cyberpsychology, behavior and social networking

"mHealth*" OR "m-health*" OR "mobile health" OR "mobile device" OR ((mobile OR smart OR tablet) NEAR/5 (app* OR application* OR phone*)) OR "mobile app" OR "smartphone" OR "mobile phone" AND ("depress*" OR "self – harm" OR suicid* OR anxi* OR "PTSD" OR "social anxi*" OR "separation anxi*"OR phobia OR "generalised anxiety disorder" OR "OCD" OR "conduct disorder" OR "eating disorder" OR anorexi*OR bulimi* OR "binge eating" OR "body image" OR "mental health*" OR Schizophren* OR "bipolar affective disorder" OR psychos* OR "insomnia*" OR stress*) AND (child* OR teenage* OR adolescen* OR "young per*" OR youth* OR "young adult*") Search limits dates Jan 2008 – Dec 2016; Language – English.

Internet Interventions

"mHealth*" OR "m-health*" OR "mobile health" OR "mobile device" OR ((mobile OR smart OR tablet) NEAR/5 (app* OR application* OR phone*)) OR "mobile app" OR "smartphone" OR "mobile phone"

Hand searched volumes 1 – 4.
